# Comparison of Stored Umbilical Cord Blood and Adult Donor Blood: Transfusion Feasibility

**DOI:** 10.5505/tjh.2012.94547

**Published:** 2012-10-05

**Authors:** Rola Sahyoun Tokan, Saadet Arsan, Ömer Erdeve, Nuri Solaz, Aslıhan Avcı, Serenay Elgün Ülkar, Elif Gülyapar, Zeynep Üstünyurt, Zeynep Bıyıklı, Sabri Kemahlı

**Affiliations:** 1 Ankara University, Faculty of Medicine, Department of Pediatrics, Ankara, Turkey; 2 Ankara University, Faculty of Medicine, Department of Pediatrics, Division of Neonatology, Ankara, Turkey; 3 Ankara University, Faculty of Medicine, Department of Pediatrics, Division of Pediatric Hematology, Ankara, Turkey; 4 Ankara University, Faculty of Medicine, Department of Biochemistry, Ankara, Turkey; 5 Ankara University, Faculty of Medicine, Department of Biostatistics, Ankara, Turkey; 6 Ankara University, Faculty of Medicine, Serpil Akdağ Blood Center, Ankara, Turkey; 7 Dr. Zekai Tahir Burak Women’s Hospital, Ankara, Turkey

**Keywords:** Transfusion, newborn, VLBW preterm, Umbilical cord blood, Blood storage

## Abstract

**Objective:** This study aimed to compare the storage properties of red blood cell (RBC) concentrates of umbilical cordblood (UCB) and adult donor blood (ADB), and to evaluate the feasibility of UCB-RBC concentrate as an autologoussource for blood transfusion in very low birth weight (VLBW) preterm neonates.

**Material and Methods:** In all, 30 newborn (10 preterm, 20 full term) UCB and 31 ADB units were collected.RBC concentrates were stored and compared with regard to pH, potassium (K^+^), 2,3-biphosphoglycerate (2-3-BPG),adenosine tri-phosphate (ATP), plasma Hb, and bacterial contamination on d 1, 21, and 35 of storage.

**Results:** The K^+^ level increased with time and differed significantly between storage d 1 and 21, and between storaged 1 and 35 in both the UCB and ADB units. Initial and d 21 K^+^ levels were higher in the UCB units than in the ADBunits. The 2,3-BPG level did not differ significantly between the UCB-PRC and ADB-PRC samples. After 35 d of storageboth UCB-PRC and ADB-PRC samples exhibited significant differences from the initial free Hb, intracellular ATP, andpH values. Significant differences in intracellular ATP and pH were also observed between the UCB-PRC and ADB-PRCsamples.

**Conclusion:** The volume of harvested and prepared UCB-PRC can be used for some of the blood transfusions requiredduring the neonatal period and thus may decrease the number of allogeneic transfusions, especially in preterm newborns.The hematological and biochemical changes that occurred in UCB during storage were comparable with those observedin ADB, and do not pose a risk to the immature metabolism of neonates. UCB-RPC prepared and stored under standardconditions can be a safe alternative RBC source for transfusions in VLBW newborns.

## Abstract (tr)

**Amaç:** Çalışmada kordon kanı ile erişkin bağışçı kanlarından elde edilen eritrosit süspansiyonlarının özelliklerini ve saklama sırasında oluşan değişiklikleri izleyerek, kordon kanından elde edilen eritrosit süspansiyonlarının çok düşük doğum ağırlıklı yenidoğanlarda otolog bir transfüzyon kaynağı olarak kullanılabilirliğini değerlendirmek amaçlanmıştır.

**Gereç ve Yöntemler:** 30 yenidoğan (20 preterm, 10 term) kordon kanı ile 31 erişkin bağışçı kanı toplandı. Hazırlanan eritrosit süspansiyonları standard koşullarda saklanarak 1., 21. ve 35. günlerde pH, potasyum (K+), 2,3-BPG, adenozin trifosfat (ATP), plazma Hb ve bakteriyel kontaminasyon bakımından incelendi.

**Bulgular:** Potasyum değerlerinin zamanla artarak, hem kordon kanı hem de erişkin kanlarında 1.-21. ve 1-35. günler arasında anlamlı farklar saptandı. İlk ve 21. Gün değerlerinin kordon kanında erişkin kanlarından daha yüksek olduğu saptandı. 2,3-BPG değerleri kordon kanı ve erişkin kanlarında anlamlı fark göstermedi.Saklamanın 35. gününde hem kordon kanı hem de erişkin kanlarında serbest Hb, ATP ve pH değerlerinin ilk değerlere gore anlamlı fark gösterdiği belirlendi.Bu parametreler açısından kordon kanı ve erişkin kanı grupları arasında da fark olduğu görüldü.

**Sonuç:** Toplanan ve hazırlanan kordon kanı eritrosit süspansiyonları yenidoğan dönemindeki transfüzyon gereksinimini kısmen karşılayabilir ve böylece allogeneik transfüzyonları azaltabilir. Saklama sırasında oluşan hematolojik ve biyokimyasal değişiklikler erişkin bağışçı kanlarındakilere benzerlik göstermekte olup yenidoğan metabolizması için bir risk oluşturacak düzeyde değildir. Kordon kanından hazırlanıp standart koşullarda saklanan eritrosit süspansiyonları çok düşük doğum ağırlıklı yenidoğanlar için bir seçenek olabilir.

## INTRODUCTION

The majority of neonates usually have uncomplicated hematological adaptation to their physiological circumstances following delivery; however, very premature neonates are prone to suffer from mild to severe anemia, which must be corrected. At present, adult donor blood (ADB) is used for neonates that require transfusion [1,2,3]. Although the risk of transfusion-associated infections has been significantly reduced due to changes in blood collection, testing, processing, and storage, the risks associated with blood transfusion have not been reduced to zero and these risks are well documented [4,5]. 

Umbilical cord blood (UCB) is emerging as an option for autologous red cell transfusion in low-birth-weight (LBW) and preterm infants. Its autologous nature negates the risks inherent in transfusion from adult donors; however, few studies have compared the properties of red blood cell (RBC) concentrates from UCB and ADB, and the changes that occur in each during storage. The aim of the present study was to compare the storage properties of UBC and ADB RBC concentrates, and to evaluate the feasibility of UBC RBC concentrates as an autologous source of blood transfusion in very low birth weight (VLBW) preterm neonates.

## MATERIALS AND METHODS

**Participants**

ADB samples were collected at Ankara University, Serpil Akdağ Blood Center, Ankara, Turkey, from 31 healthy male and female donors that met the standard blood donor criteria and provided informed consent to participate in the study. The donors were tested to rule out hepatitis B, hepatitis C, HIV, and syphilis infection. UCB was collected at Zekai Tahir Burak Women’s and Maternity Hospital, Ankara, Turkey, from 20 full-term and 10 preterm infants (gestational age <32 weeks) that were delivered via caesarean section; informed consent was obtained from their parents before delivery. 

Exclusion criteria for the collection of UCB were maternal viral or bacterial infections, including suspected chorioamnionitis (defined by the presence of purulent amniotic fluid, C-reactive protein [CRP] >10 mg L–1, or an immature to total white blood cell (I:T) ratio in the mother >0.15). None of the blood units tested in this study were used for transfusion. 

The study protocol was approved by the ethics committees of Ankara University, School of Medicine and Zekai Tahir Burak Women’s and Maternity Hospital, and was performed in accordance with the Declaration of Helsinki. 

**Blood collection**

Standard units (450±45 mL) of blood were drawn from each adult donor into a main blood bag that contained 63 mL of citrate-phosphate-dextrose (CPD). Pediatric blood bag sets (Kansuk Laboratuarı, Istanbul Turkey) were used for UCB collection. As standard blood bags conÖzet pretainCPD solution at a ratio of 1:7 for 450 mL of wholeblood, the volume of CPD for use in UCB collection bagswas calculated using the same ratio and an estimated maximumUCB volume of 140 mL; thus, UCB was collectedinto bags containing 21 mL of CPD.

Placental blood collection was performed using a specificallymodified placental blood collection system. Beforeplacental blood collection the blood collecting system’sprimary bag was placed approximately 1 m below the levelof the placenta. Placental blood was collected immediatelyafter birth, with the placenta in utero. Cord clamping wasperformed immediately after the baby was born and thenthe puncture site was identified approximately 10 cm fromthe placenta-cord interface. The umbilical cord vein wasthen punctured with a cannula attached at the longer collectiontube of the collection system. The collecting tubewas clamped before the blood clotted and the blood flowstopped. Collected blood was immediately transported tothe blood bank where it was separated into blood components,as RBCs and plasma.

**Separation and RBC unit preparation**

Collected UCB was stored under standard storage conditions(2-6 °C) and processed within 24 h of collection.In all, 30 newborn (10 preterm and 20 full-term) UCB and31 ADB units were collected. UCB and ADB units werecentrifuged at 2600 g for 5.5 min. Whole blood was thenseparated into plasma and buffy coat depleted RBCs usinga blood component separation device. The total collectedvolume of UBC packed red cell concentrate (UCB-PRC)and 31 ADB-PRC samples were recorded. Then, 8 mL ofblood from the main bag was transferred to each of 2 accessorybags. Blood samples for testing were withdrawn fromthe accessory bags to avoid contamination of the main bagon d 21 and d 35. All blood units were stored in the sameblood bank refrigerator at 4 °C. 

**Biochemical tests**

 Biochemical (2,3-biphosphoglycerate [2,3-BPG], adenosine triphosphate [ATP], free Hb, K+, and pH) parameters were measured in all UCB-PRC samples on d 1, 21, and 35 of storage. Data were compared with data obtained from 31 ADB-PRC samples stored under the same conditions. Microbiological testing was performed on all samples via blood cultures using the Bactec system. The recommended blood volume (2 mL) was injected into 50-mL Bactec NR 660 aerobic and anaerobic pediatric blood culture bottles (Becton Dickinson, Franklin Lakes, NJ, USA). Inoculation of the samples was performed every week. Cultures were assessed daily for the presence of bacterial growth for 1 week after inoculation. Serum K+ was measured via ion-selective electrode (ISE Olympus AU 600, Hamburg, Germany) 

**ATP Measurement**

 Intracellular ATP was measured via bioluminescence assay. The test principle is based on luciferase from Photinus pyralis (American firefly) catalyzing the following reaction: ATP+D-luciferin+O_2_→oxyluciferin+PPi+AMP+ CO_2_ + light. 

The methods specified in the commercial kit (ATP Bioluminescence Assay Kit CLS II, Roche Cat. No. 1699695) were used. 

**2,3-BPG measurement**

 2,3-BPG was measured spectrophotometrically based on the change in absorbency at 340 nm caused by oxidation from NADH to NAD due to glycerol-3-phosphate dehydrogenase (GDH), according to the methods specified in the commercial kit (2,3-biphosphoglycerate Kit, Roche, Cat. No.10148334001). 

**Free Hb measurement**

 Free Hb was measured spectrophotometrically, as described previously [6]. 

**Statistical analysis**

 Data analysis was performed using SPSS v.11.5. As thevariables 2,3-BPG, ATP, K+, and pH were not normallydistributed, Friedman’s ANOVA followed by Wilcoxontest with Bonferroni correction was used to determine thedifferences within groups, based on time. The differencesof measured variables between UCB and ADB was analyzedusing the non-parametric Mann-Whitney U test withBonferoni correction [7].

In that case 3 different tests were performed to reducethe error made by Bonferroni correction and thus, P<0.05/3 = P<0.017 was considered statistically significant;when analyzing differences between 2 different timepoints, this level was accepted as P<0.05/2 = P<0.025,as 2 different tests were performed.

## RESULTS

**Characteristics of the collected and processed blood**

The volume of UCB collected ranged between 43 and94 mL, depending on gestational age. The harvested UCBvolume was 43-77 mL (mean: 57.1 0.5 mL) for pretain term newborns and 62-94 mL (mean: 77.8 ± 10.5 mL) for full-term newborns. Birth weight was associated with the volume of collected UCB; approximately 24.8 mL of UCB kg–1 of bodyweight was collected, irrespective of gestational age. Mean RBC volume, on the other hand, was 14.6±2.4 mL kg–1 for the preterm newborns and 12.6±1.5 mL kg–1 for the full-term newborns. 

**Microbial contamination**

 Bacterial contamination was not observed in any of the UCB or ADB units during storage. 

**Changes during storage**

 Storage parameters for the 30 UCB-PRC and 31 ADBPRC samples are shown in Table 1 Table 2. The K^+^ level increased with time in both groups, with significant differences between d 1 and 21, and between d 1 and 35. Initial and d 21 levels were higher in the UCB samples than in the ADB samples ([Table t3]Table 3). The 2,3-BPG level did not differ significantly between UCB-PRC and ADB-PRC samples (P>0.017); however, there was a significant decrease (P<0.01) on d 21 and d 35—as compared to d 1—in both groups (Table 4). On d 35 of storage free Hb (Table 5), pH (Table 6), and intracellular ATP values (Table 7) differed significantly from the initial values in both UCB and ADB RBCs. Significant differences in these parameters were also observed between UCB-PRC and ADB-PRC.

## DISCUSSION

The present study aimed to evaluate changes in UCBPRC and ADB-PRC in an additive storage medium (CPD) during storage. This preclinical study was considered to be essential for determining the feasibility of the clinical use of UCB. No transfusions were performed with the blood units collected during this study; however, the use of UCB for transfusion has been studied previously. Tamayo [8] reported the successful transfusion of 12 unfractionated UCB specimens (volume: 25-150 mL) in 1966 and suggested that UCB could be used instead of allogeneic transfusion. Ballin et al. published a case report describing how transfusion of 2 units of autologous UCB in a preterm baby did not cause any side effects [9]. In addition to the publication by Anderson et al. [10], Strauss published a warning on the clinical use of UCB, in which he suggested that the relative risks due to infections, changes during storage, and efficacy—as compared to allogeneic transfusions—should be clarified before the start of clinical studies[11].

Eichler et al. studied VLBW infants (body weight<1000 g) and collected a net mean UCB volume of only 37mL; RBC preparation was successful only in exceptionalcases. They did conclude that the preparation of autologousRBCs from UCB collected from preterm infants wastechnically possible [12]. One of the concerns of UCB hasbeen the small volume of blood collected. Researchershave tried to show the correlation between the volume ofcollected blood and birth weight, type of delivery (vaginal or caesarean section), and collection method (ex utero versus in utero) [12,13,14,15,16,17,18,19. Despite some inconsistent results, Surbek et al. [14,15], Pafumi et al. [16,17], and Solves et al. [19] reported that in utero collection after caesarean delivery yielded higher collection, [16,17,18,19]. Despite some inconsistent results, Surbek et al. [14,15], Pafumi et al. [16,17], and Solves et al. [19] reported that in utero collection after caesarean delivery yielded higher collection volume. As such, in utero collection was employed in the present study. 

Some studies examined the correlation between the volume of collected blood, and birth weight and gestational age of newborns. Garritsen et al. and Brune et al. harvested approximately 20 mL kg–1 of bodyweight, independent of birth weight, using both in utero and ex utero collection [20,21]. Eichler et al., on the other hand, reported contamithat there was an inverse correlation between the volume of collected blood, and gestational age and birth weight. They harvested 43 mL kg–1 of body weight from preterm infants [12]. Surbek et al. collected a median volume of 21 mL (range: 8-38 mL) following delivery of infants with a gestational of 22-32 weeks and a median volume of 49 mL (range: 21-103 mL) after delivery of infants with a gestational age of 33-36 weeks; birth weight and the volume of collected blood were not correlated [14,15]. Jansen et al. reported a UCB collection volume of 23 mL kg–1 in infants with a gestational age <32 weeks, but did not note a correlation between the volume of harvested UCB, and gestational age, birth weight, placental weight, or method of delivery [22]. Khodabux et al. observed a correlation between the collected blood volume and gestational age. The collected volume of UCB was 32±7.7 mL for infants with a gestational age of 24-28 weeks, 44±27.4 mL for those with a gestational age 28-30 weeks, and 33±13.3 mL for infants with gestational age of 30-32 weeks. The volume of harvested UCB was 16±15 mL kg–1 in infants with a birth weight <1000 g, 18 ± 18 mL kg–1 for those weighing 1000-1250 g, and 20±23 mL kg–1 for those with a birth weight >1250 g; however, birth weight and the volume of harvested blood were not correlated [23]. Mean volume of harvested UCB in the present study was 57.11±10.46 mL in the preterm infants and 77.84±10.54 mL in the full-term infants, and was 24.87 mL kg–1 of bodyweight, irrespective of gestational age. Mean RBC volume, on the other hand, was 14.63±2.43 mL kg–1 in the preterm infants and 12.55±1.46 mL in the full-term newborns, which is similar to previous reports. Mean UCB volume kg–1 of birth weight was higher in the preterm infants; however, correlation analysis between the collected volume, and birth weight and gestational age was not performed. 

Numerous studies reported that K^+^, 2,3-BPG, ATP, and pH values in stored blood and blood components change with time, and research has been conducted in an effort to increase the quality of stored blood [24,25,26,27,28,29,30]. Studies on the biochemical and hematological properties of stored UCB, however, are less common,[26,27,28,29,30]. Studies on the biochemical and hematological properties of stored UCB, however, are less common. Garritsen et al. studied whether or not UCB-PRC could be used as an alternative to ADB-PRC. They developed a system for collecting and preparing UCB-PRC, and measured standard storage parameters during 35 d of storage in extended storage medium (SAG mannitol). The initial laboratory UCB-PRC parameters were similar to those of ADB-PRC. After 35 d of storage UCB-PRC had a hemolysis rate 1% higher than that of ADB-PRC and a significant decrease in ATP. UCB PRC met the same quality criteria as ADB-PRC after 35 d of storage [20]. 

Bifano et al. studied ‘placental whole blood’ stored in CPDA for 28 d and reported that the K+ level on d 28 was comparable to that in stored ADB. In the UCB ATP, 2,3-BPG, and pH decreased, and some morphological changes, an increase in osmotic fragility, and a minimal increase in hemolysis were noted. They also reported that fetal erythrocytes were preserved better in citrate-phospohate- adenine-1 solution (CPDA-1) than in CPD [31]. The hemolysis rate, however, was higher in the studies by Brune et al. [32], Garritsen et al. [20], and Widing et al. [33]. In a recent review Khodabux and Brand reported that the preservative solution used, not the manipulation of UCB, might contribute to storage damage. This has been suggested by some other studies that reported better results using the storage solution PAGGSM that contained additional phosphate and guanosine than those obtained using SAG-M [34]. 

In the present study the 2,3-BPG level did not differ significantly between UCB-PRC and ADB-PRC (P > 0.017). After 35 d of storage, free Hb, intracellular ATP, and pH values were significantly different than initial values in both UCB RBCs and ADB RBCs. Additionally, it was observed that 2,3-BPG, ATP, and pH values decreased in both blood groups, whereas K+ increased. These significant differences in both blood groups were thought to have been due to an increase in hemolysis and decrease in intracellular ATP. The higher K+ value on d 1 of storage reached a similar level in ADB on d 35. Bacterial contamination has been one of the concerns of the groups dealing working with UCB. In the present study bacterial contamination was not observed in any of the stored UCB-PRC samples. Eichler et al. had raised concerns with regard to the high rate of bacterial contamination [12]. Garritsen et al. [20] reported a bacterial contamination rate of 1.84% in 390 collected UCB units, and Jansen et al. reported there was bacterial contamination in 6 of 91 (7%) collected UCB units stored for 30 days [22]. 

In conclusion, the volume of harvested and prepared UCB-PRC is similar to previous studies and can supply for some of the blood transfusions required in neonatal period and thus, its use may decrease number of allogeneic transfusions, especially in premature newborns. Hematological and biochemical changes that occur to UCB during storage are comparable to those observed in ADB and do not pose a risk to the relatively immature metabolism of neonates [35,36,37]. The lack of any observable bacterial contamithat nation in the present study indicates that the collectionmethod employed was reliable. The present findings showthat the collection, separation, and storage of UBC-PRC,and the changes that occur during 35 d of storage can yielda product that is feasible for use under clinical conditions.In addition, autologous transfusion is safer than allogeneictransfusion, in terms of immunohematology and infectionrisk. The use and outcomes of UCB however, remain to bedetermined by further clinical studies.

Abbreviations: VLBW: Very low birth weight, UCB:Umbilical cord blood, ADB: Adult donor blood, RBC: RedBlood Cell, K^+^: Potassium, 2,3-BPG: 2,3-biphosphoglycerate,ATP: Adenosine tri-phosphate, UCB-PRC: Umbilical cordblood- packed red blood cell concentrate, A-PRC: Adultpacked red blood cell concentrate, CPD: Citrate-phosphatedextroseadditive solution

This study has been supported by Ankara UniversityScientific Research Projects Fund

## Figures and Tables

**Table 1:**
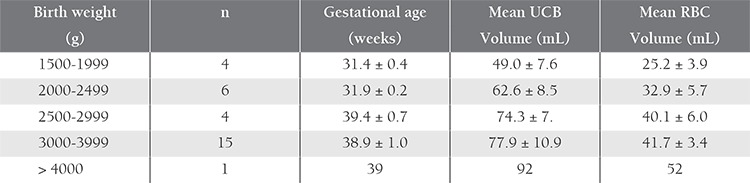
Characteristics of the neonates and UCB volume.

**Table 2:**
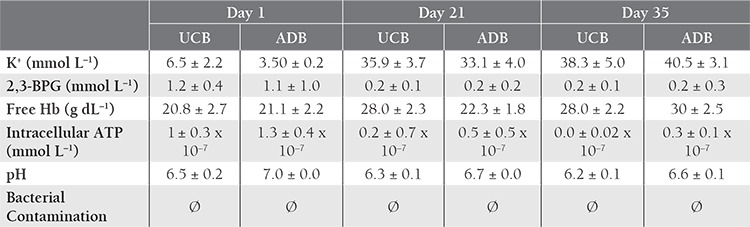
Mean hematological and biochemical parameters.

**Table 3 t3:**
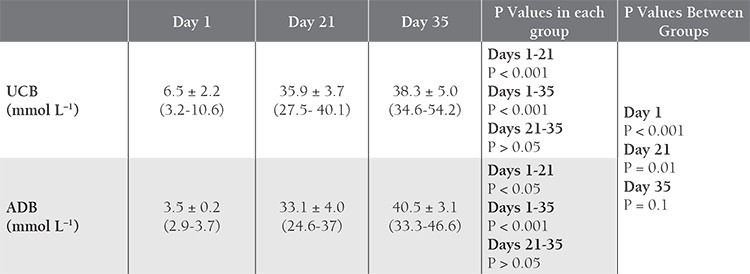
Comparison of K+ values in UCB and ADB samples over time.

**Table 4:**
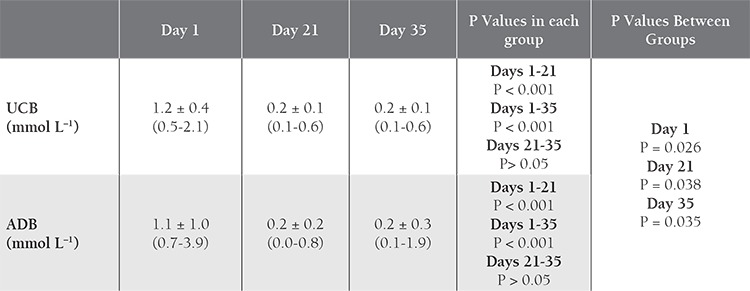
Comparison of 2,3,-BPG values in UCB and ADB samples over time.

**Table 5:**
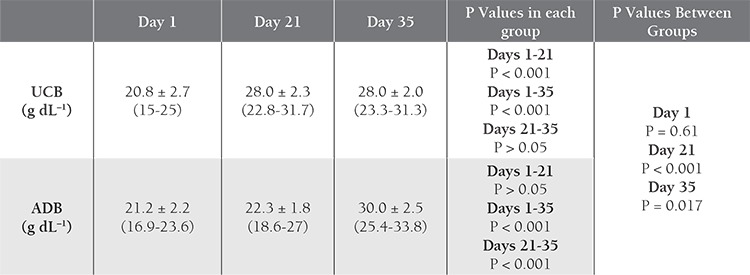
Comparison of free Hb values in UCB and ADB samples over time.

**Table 6:**
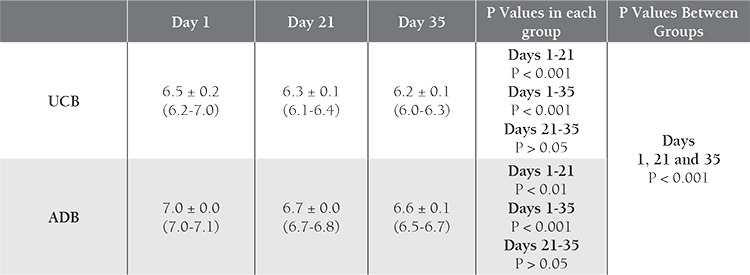
Comparison of pH values in UCB and ADB samples over time.

**Table 7:**
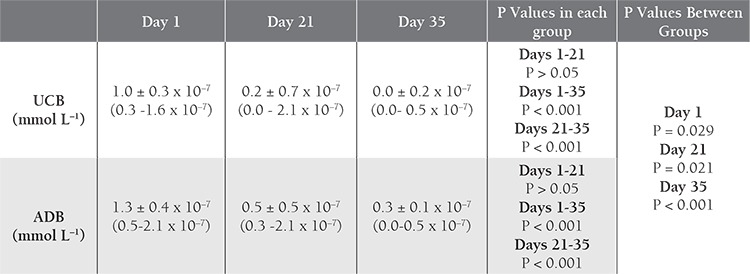
Comparison of ATP values in UCB and ADB samples over time.
